# A Practical Treatise on the Diseases of Children

**Published:** 1850-05

**Authors:** 


### ARTICLE IV.

*A Practical Treatise on the Diseases of Children.*—By D. Francis Condie, M.D., Secretary of the College of Physicians; Member of the American Medical Association; Member of the American Philosophical Society; Honorary Member of the Philadelphia Medical Society, etc. Third Edition. Revised and Augmented. pp. 696: oct: Philadelphia: Lea & Blanchard: 1850. (From the Publishers, by Express.)

This standard work upon the Diseases of Children is improved in this edition by a careful revision by the author, and the addition of an entire chapter upon Epidemic Meningitis, "a disease which, although not confined to children, occurs far more frequently in them than in adults."

We have no hesitation in saying it is one of the very best works upon the subject extant. This edition is got up in remarkably good style, quite worthy of the high reputation of the publishers. E.

